# Untargeted Lipidomics Reveals Characteristic Biomarkers in Patients with Ankylosing Spondylitis Disease

**DOI:** 10.3390/biomedicines11010047

**Published:** 2022-12-25

**Authors:** Zhengjun Li, Wanjian Gu, Yingzhuo Wang, Bin Qin, Wei Ji, Zhongqiu Wang, Shijia Liu

**Affiliations:** Affiliated Hospital of Nanjing University of Chinese Medicine, #155 Hanzhong Road, Qinhuai District, Nanjing 210029, China

**Keywords:** lipidomics, ankylosing spondylitis, biomarkers, disease activity, UPLC-Q-Exactive

## Abstract

**Objective.** Ankylosing spondylitis (AS) is a chronic inflammatory disease of the axial skeleton. Early and accurate diagnosis is necessary for the timely and effective treatment of this disease and its common complications. Lipid metabolites form various kinds of bioactive molecules that regulate the initiation and progression of inflammation. However, there are currently few studies that investigate the alteration of serum lipid in AS patients. **Methods.** Blood samples were collected from 115 AS patients and 108 healthy controls (HCs). Serum-untargeted lipidomics were performed using ultrahigh-performance liquid chromatography coupled with Q-Exactive spectrometry, and the data were determined by multivariate statistical methods to explore potential lipid biomarkers. **Results.** Lipid phenotypes associated with disease activity were detected in the serum of patients with AS. Of all 586 identified lipids, there are 297 differential lipid metabolites between the AS and HC groups, of which 15 lipid metabolites are significant. In the AS groups, the levels of triacylglycerol (TAG) (18:0/18:1/20:0) were increased, and the levels of phosphatidylcholine (PC) (16:0e/26:4) and PC (18:1/22:6) were decreased. The areas under the receiver operating characteristic curve (AUC) of TAG (18:0/18:1/20:0), PC (16:0e/26:4), and PC (18:1/22:6) were 0.919, 0.843, and 0.907, respectively. **Conclusion.** Our findings uncovered that lipid deregulation is a crucial hallmark of AS, thereby providing new insights into the early diagnosis of AS.

## 1. Introduction

Ankylosing spondylitis (AS) is a form of chronic inflammatory arthritis characterized by back pain in young adults, and it can lead to severe structural damage and deficiency in the performance of daily activities [1]. AS usually impairs the sacroiliac joints and spine. Patients can experience progressive stiffness and pain, which has led to the consideration of this disease as a global public health issue [2]. Since the early stage of AS often has no obvious symptoms aside from unexplained back pain, the timely diagnosis of AS is very difficult. According to estimates, it takes 5 years from the onset of back pain to arrive at a definite diagnosis of AS. This delay in treatment may ultimately contribute to the poor outcomes of these patients. Therefore, early diagnosis is of utmost importance for interventions in the progression of AS. In addition, AS can also affect several other systems, such as the eyes, skin, gastrointestinal system, heart, and vascular structures [3]. For example, it is known that the incidence of cardiovascular disease, including atherosclerosis, is increased in AS patients, and that this cardiovascular disease partly results in the mortality of AS patients. However, the underlying mechanism of the increased cardiovascular morbidity has not been fully understood. Currently, the diagnosis of AS is based on the comprehensive evaluation of clinical symptoms and radiological changes [4]. The use of serial assessments of radiation observations to evaluate disease progression is often discouraged. The Bass Ankylosing Spondylitis Disease Activity Index (BASDAI) and Ankylosing Spondylitis Disease Activity Score (ASDAS) are the two gold-standard measures used to evaluate the disease activity and status of AS. The BASDAI involves six aspects, including fatigue, back pain, peripheral joint pain/swelling, enthesitis, and morning stiffness [5]. The ASDAS includes the self-reported back pain index, the duration of morning stiffness, pain/swelling of peripheral joints, and a global assessment of disease activity [6]. Although some autoimmune antibodies have been successfully applied in the clinical diagnosis of early rheumatoid arthritis, potential diagnostic biomarkers in AS including immunoglobulin G (IgG) and IgA were not specific and selective [7]. Many experts also consider that several inflammatory factors can predict the initiation of AS, such as C-reactive protein (CRP), a marker of systemic inflammation. This marker positively correlates with the progression of sacroiliitis in AS patients, but whether it can be generally used is still unknown [8,9]. In addition, human leucocyte antigen B27, interleukins 17 and 23, matrix metalloproteinases, and noncoding RNAs in serum have been recently found to highly associate with the etiology of AS, but neither of them can serve as a good candidate for a diagnostic marker alone [7,10,11]. Metabolites are end products of biological processes and their concentrations can reflect disease states and pathological conditions [12]. Several studies have used metabolomics to identify the biomarkers of disease diagnosis and activity in serum and urine from patients with AS. These studies have highlighted the differences in metabolites such as glycine, tryptophan, and butyrate, but the sample size of most of these studies is small and the related lipid profiles have already been widely revealed [2,13,14]. During the progression of AS, systemic inflammation is thought to lead to changes in the lipid profile. The emergence of systemic inflammation can cause the release of free oxygen radicals, which accelerate the abnormalities of the lipid profile. Additionally, among various kinds of lipids, sphingolipids, resolvins, and protectins are key inflammation regulators, and some other lipids such as glycerolipids and lipid mediators also participate in the crosstalk between inflammation and metabolism.

Lipidomics is a new approach used to systematically analyze the cellular lipidome. By comparing the changes in the lipid metabolic networks in different pathological and physiological states, this approach can be used to identify the key lipid biomarkers in metabolic regulation and, ultimately, reveal the mechanisms of lipids in various biological activities. In this study, ultrahigh-performance liquid chromatography coupled with hybrid quadrupole orbitrap mass spectrometry were used to analyze the serum of 115 patients with AS and 108 healthy controls (HCs). Consequently, it was determined that the expansion of the sample size could make the results more convincing. We aimed to study the changes in systemic lipid metabolism in patients with AS and to find potential serum markers for the diagnosis of AS.

## 2. Methods

### 2.1. Study Populations

All procedures were performed in compliance with the Helsinki Declaration and approved by the Institutional Review Board and the Ethics Committee of the First Affiliated Hospital of Nanjing University of Traditional Chinese Medicine (2018NL-106-02). After reviewing a written plan of the whole study, all volunteers signed and provided written informed consent.

A total of 223 participants were recruited between September 2017 and January 2019, and all the participants were Asian. A total of 115 patients with newly diagnosed AS were recruited from the Affiliated Hospital of Nanjing University of Chinese Medicine. The patients did not receive any physical or medicinal treatment (Nonsteroidal anti-inflammatory drugs (NSAIDs), conventional synthetic disease-modifying antirheumatic drugs (csDMARDs), and biologic disease-modifying antirheumatic drugs (bDMARDs), etc.), and they had neither chronic diseases nor comorbid disorders such as rheumatism, non-alcoholic fatty liver disease, atherosclerosis, or pancreatitis. AS diagnoses were conducted according to the criteria of Assessment of Spondyloarthritis International Society (ASAS) with X-ray changes. Patients with other rheumatic diseases, systemic diseases, or tumors were excluded from this study. A total of 108 healthy people from the physical examination center were selected as the HC group. All HCs had no history of chronic disease or rheumatism. Demographic and clinical parameters as well as laboratory indicators such as HLA-B27, CRP, ESR, alkaline phosphatase (ALP), and albumin (ALB) were recorded (Table 1). In this study, the exclusion criteria included (1) cardiovascular and cerebrovascular, liver, kidney, hematopoietic system, and other serious primary diseases, as well as complications including tumors, diabetes, or hypertension; (2) pregnancy or breastfeeding women; (3) patients undergoing physical and/or medicinal treatment; and (4) subjects suffering from external infection.

### 2.2. Sample Preparation

Whole-blood venous samples (3 mL) were collected from the patients using a disposable vacuum blood collection tube containing a procoagulant and separation gel. After the blood samples had clotted for 30 min, the serum was separated by centrifugation at 3000 rpm for 10 min within 2 h.

The samples were thawed on ice. Then, 225 μL of ice-cold methanol containing a mixture of internal standards was added to 40 μL of serum, and the mixture was vortexed for 10 s. Then, 750 μL of cold methyl tert butyl ether was added, and the mixture was vortexed for 10 s and shaken on an orbital mixer at 4 °C for 10 min. After the addition of 188 µL of room-temperature LC/MS-grade water, the mixture was vortexed for 20 s and centrifuged for 2 min (14,000 rcf, 4 °C). The supernatant was transferred to clean, new tubes and dried in a vacuum centrifuge. The upper phase lipids were reconstituted with 110 μL of methanol:toluene (9:1) for UPLC-QE MS analysis. Untargeted lipidomic analysis was performed using the Dionex UltiMate 3000 Ultra-Performance Liquid Chromatography (UPLC) system (Santa Clara, CA, USA) coupled with an electrospray ionization source with a Q Exactive mass spectrometer (Thermo Fisher Scientific, USA). For the separation of lipids, 1 µL of sample solution was injected into a reversed-phase Waters Acquity UPLC CSH C18 column (100 mm × 2.1 mm, 1.7 μm) maintained at 60 °C by gradient elution. All MS experiments were performed in positive and negative ion modes using a heated ESI source with a spray voltage of 3 kV (positive). To monitor the robustness and stability of analytical method, quality control (QC) samples were prepared by pooling 20 µL of serum from each sample and were analyzed once per batch of 10 samples. The orders of sample preparation and injection were both randomized to avoid systematic biases.

### 2.3. Data Analysis

The raw data were preprocessed by MS-DIAL; then, the variables were identified by the Fiehn Lab database. After removal of the features with missing values > 80%, the data were normalized by MetaboAnalyst 5.0 (https://www.metaboanalyst.ca/), accessed on 1 November 2020. The preprocessed data were uploaded to SIMCA-P version 14.1 (Umetrics, Umeaa, Sweden) for multivariate statistical analysis, including principal component analysis (PCA) and orthogonal partial least-squares discriminant analysis (OPLS-DA). The variable importance in the projection (VIP) was obtained from the OPLS-DA model. Univariate statistical tests were performed on SPSS version 25.0 (IBM, Armonk, NY, USA). The false discovery rate (FDR) method of Benjamini–Hochberg was used to correct the Wilcoxon rank-sum test. Lipids that met the criteria of VIP > 1.0, *p* value < 0.05, and FDR < 0.05 were considered significantly different.

To further identify potential diagnostic biomarkers from the differentially abundant lipids, the forward stepwise binary logistic regression method and the Wald test were used to establish a classification model using SPSS software. Then, the diagnostic efficacy was evaluated by receiver operating characteristic (ROC) curve analysis, and the area under the ROC curve (AUC) was calculated.

## 3. Results 

### 3.1. Basic Characteristics of the Participants

In this study, a total of 223 serum samples from 108 HCs and 115 patients with AS were collected to identify candidate biomarkers. The demographic characteristics and clinical information of the subjects are shown in Table 1.

### 3.2. Global Lipid Shifts in AS

**Figure 1:**
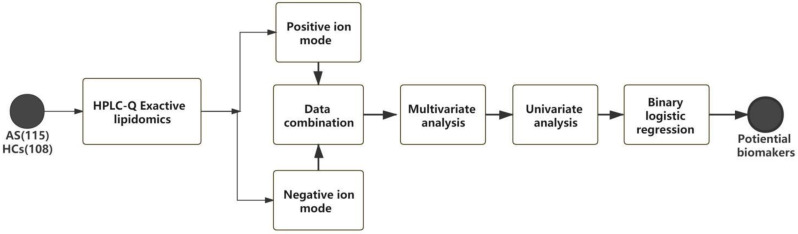
Design of this study.

**Figure 2:**
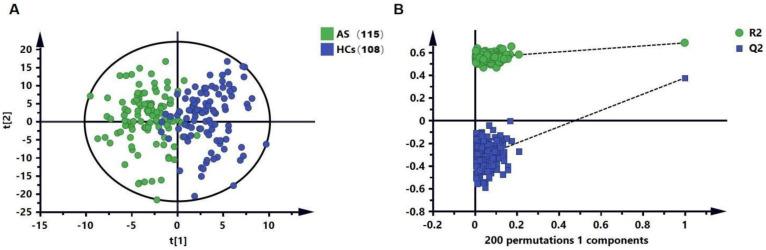
Identification of potential lipid biomarkers for the diagnosis of AS. (**A**) Orthogonal partial least squares discriminant analysis (OPLS-DA) score plot based on HC and AS groups in the dis-covery set. (**B**) Results of 200 permutation tests of the OPLS-DA model.

The workflow of this study is shown in Figure 1. In the untargeted lipid metabolomics analysis, we examined 223 serum samples. The lipid-profiling data were acquired in positive and negative ESI modes with two injections, and a total of 586 lipids were determined. We further applied the OPLS-DA (Figure 2A) analysis model to identify the differences in the metabolic profiles between the AS and HC groups. Without the overfitting of the model (Figure 2B), there was an apparent separation between the two groups, indicating that the lipid metabolic pattern was changed. Based on the significant changes in the comparison between the lipid metabolites of the AS and HC groups, multivariate and univariate criteria (VIP > 1, *p* value < 0.05, and FDR < 0.05) were used to identify 114 metabolites of the AS group, and these results were compared with the HC group (Figure 2B).

### 3.3. Biomarkers for Diagnosis and Progression of AS

**Figure 3:**
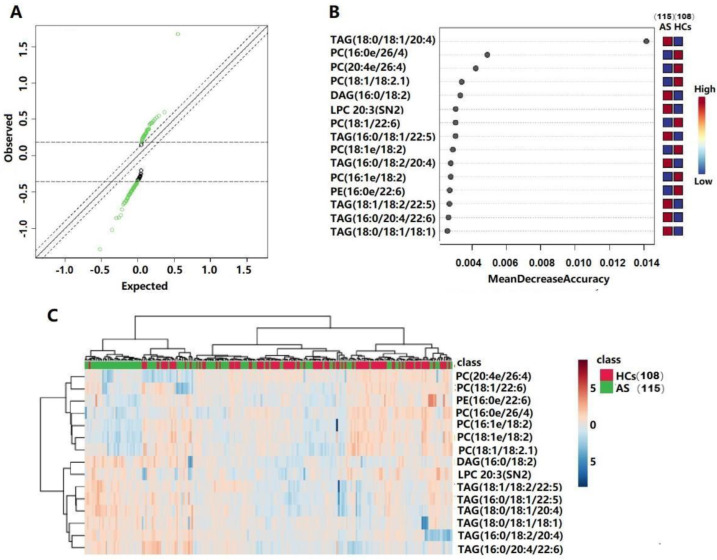
Lipidomic profiling of serum samples from 15 lipid species that distinguish HCs and AS. (**A**) The result of the SAM scatter plot of observed scores plotted versus expected scores with a delta value of 0.1. Significant lipid species are represented in green. (**B**) Significant features identified by random forest. (**C**) A heatmap of the differential lipid species between HCs and AS groups.

Significance analyses of the microarray (SAM) and random forest (RF) analyses were further used in order to screen the lipid metabolites that changed significantly during disease progression (Figure 3A,B), and 15 lipids were retained. Details of these metabolites are listed in Table 2. A heatmap showing the relative intensity distribution of these metabolites in the AS and HC groups is shown in Figure 3C.

**Figure 4:**
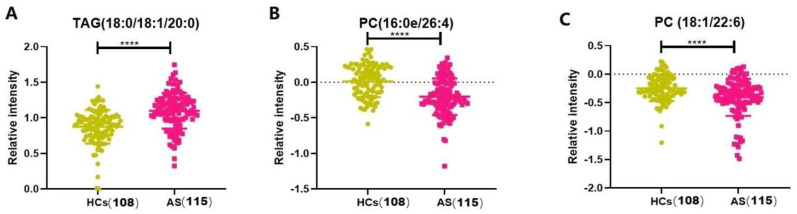
Serum-relative intensities of TAG (18:0/18:1/20:0), PC (16:0e/26:4), and PC (18:1/22:6) in the HCs and AS groups. (**A**,**B**) The levels of TAG (18:0/18:1/20:0) and PC (16:0e/26:4) were increased in the patients with AS (*p* < 0.0001). (**C**) The levels of PC (18:1/22:6) were decreased in the patients with AS (*p* < 0.0001). **** *p* < 0.001.

**Figure 5:**
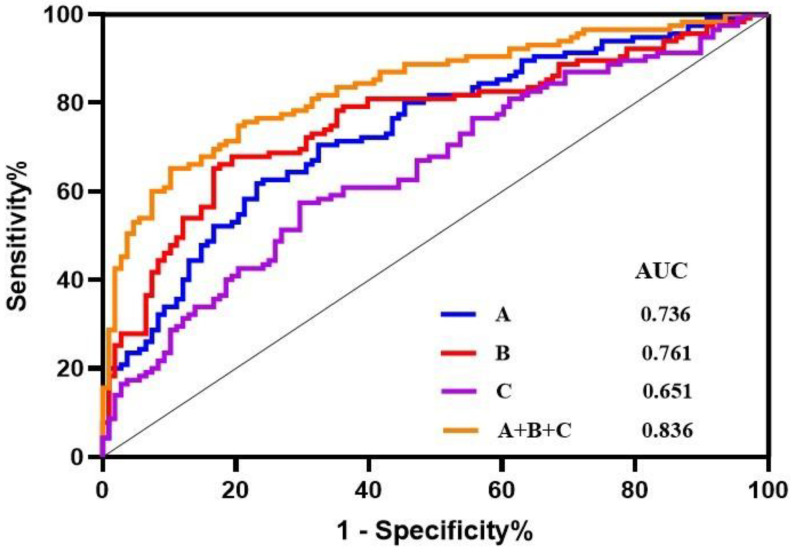
Receiver operating characteristic curve analysis (ROC) of TAG (18:0/18:1/20:0), PC (16:0e/26:4), PC (18:1/22:6), and their combination. A: PC (16:0e/26:4); B: TAG (18:0/18:1/20:0); C: PC (18:1/22:6). AUC: area under the curve.

Subsequently, a binary logistic regression was performed using SPSS 25.0 software to further analyze the 15 differentially altered lipid metabolites given above. A regression model was established using the forwarding stepwise optimization algorithm (Wald), and TAGs (18:0/18:1/20:0), PC (16:0e/26:4), and PC (18:1/22:6) were determined to be reliable lipid biomarkers. Figure 4 shows that the TAG (18:0/18:1/20:0) and PC (16:0e/26:4) levels were significantly increased, whereas the PC (18:1/22:6) levels were decreased in the patients with AS. Then, the diagnostic potential of the three lipids was evaluated (Figure 5). For the AS and HC groups, TAGs (18:0/18:1/20:0), PC (16:0e/26:4), PC (18:1/22:6), and their combination showed AUCs of 0.761, 0.736, 0.651, and 0.836; sensitivities of 65.22%, 61.74%, 57.39%, and 65.22%; and specificities of 83.33%, 76.85%, 70.37%, and 89.81%, respectively.

**Figure 6:**
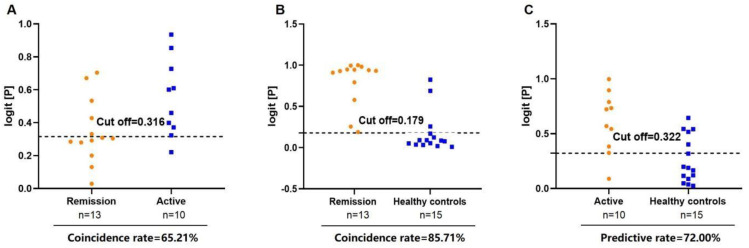
Diagnosis of disease activity in AS using the combination of TAG (18:0/18:1/20:0), PC (16:0e/26:4), and PC (18:1/22:6). (**A**) Diagnostic coincidence rate for the comparison between remission vs. active AS. (**B**) Diagnostic coincidence rate for the comparison between remission AS vs. HCs. (**C**) Diagnostic prediction rate for the comparison between active AS vs. HCs.

Finally, the correlations between the TAGs (18:0/18:1/20:0), PC (16:0e/26:4), PC (18:1/22:6), and AS disease activity were determined (Figure 6). In this study, ‘inactive disease’ was defined as ‘remission status of AS’ (<1.3), while ‘low disease activity’, ‘ moderate disease activity’, and ‘very high disease activity’ were defined as ‘Active status of AS’ (≥1.3) [15,16]. We randomly selected 23 patients who were obviously in the active or remission stage for analysis. The combination of TAGs (18:0/18:1/20:0), PC (16:0e/26:4), and PC (18:1/22:6) distinguishes remission AS from active AS and HCs, with coincidence rates of 65.21% and 85.71%, respectively. In addition, 0.322, as the cut-off value, was able to differentiate patients with active AS from HCs. Therefore, the combination of TAGs (18:0/18:1/20:0), PC (16:0e/26:4), and PC (18:1/22:6) is an ideal lipid biomarker for differentiating patients with AS from HCs. In addition, we also explored the correlation between these three lipids and CRP. The results showed that PC (16:0e/26:4) and PC (18:1/22:6) were negatively correlated with CRP (Figure S1), while TAG (18:0/18:1/20:0) was not significantly correlated with CRP. However, these results need to be verified at the molecular level.

## 4. Discussion

At present, clinical monitoring is mainly based on the X-ray diagnosis of AS, but this process is lengthy [17]. Since early AS is usually asymptomatic or has no obvious pathological changes, the early diagnosis of AS is still a difficult problem. Some biomarkers, such as immunoglobulin G (IgG), IgA, or C-reactive protein, do not achieve sufficient diagnostic sensitivity or specificity [18]. At present, omics technology has become a powerful tool for biochemical analysis, providing important insights into the processes of various diseases. As an important branch of metabolomics, lipidomics has been used in the early diagnosis of various diseases, such as Sjogren’s syndrome [19], systemic lupus erythematosus [20], and ulcerative colitis [21].In recent years, with the emergence and development of lipidomics, some researchers have tried to apply it to the study of AS. Gao P et al. analyzed the main lipid metabolism-related changes in the plasma of AS patients, and found that the AS patients presented altered concentrations of phospholipids and phosphatidylcholine in the plasma samples. The concentrations of many lipids were decreased, including linolacylglycerol phosphate choline, palmitoyllysolated phosphatidylcholine, oleylglycerol phosphate choline, and stearoylglycerol phosphate choline [22]. In our study, a nontargeted HPLC-QE serum lipid method was applied to investigate the lipid characteristics of 223 patients with AS and HCs. Consequently, it was determined that TAG (18:0/18:1/20:0), PC (16:0e/26:4), and PC (18:1/22:6) reflect the dynamic processes corresponding to the lipid levels in AS patients. These three lipids and their combinations can be used as potential biomarkers for the diagnosis of AS and to distinguish different degrees of the disease. These findings provided new insights into improving the diagnosis and treatment of AS, and further enhance our understanding of AS’s pathophysiology.

In patients with AS, the characteristics of metabolic syndrome have been found [23]. Metabolic syndrome constitutes a series of metabolic disorders, including obesity, insulin resistance, impaired glucose tolerance, hypertension, and dyslipidemia [24]. Compared with healthy people, patients with AS have higher overall mortality and cardiovascular disease (CVD)-related mortality [25,26]. Chronic inflammation, impaired lipid metabolism, enhanced lipid peroxidation, early endothelial dysfunction, and the excessive activation of coagulation cascades can lead to AS-related atherosclerotic cardiovascular disease (ACD) [27,28]. We examined the lipid profiles of patients with AS and screened them for differentially abundant lipids; consequently, we found that serum TAG (18:0/18:1/20:0) levels were increased. According to recent studies, high serum TAG and low HDL levels are considered independent risk factors for ACD [29,30]. Our results showed that TAGs (18:0/18:1/20:0) can be used as a potential biomarker for the diagnosis of AS, with a diagnostic potential of 0.761.

PC is an important component of the cell membrane, alveolar surfactants, lipoproteins, and bile, and is also the source of lipid messengers such as lysophosphatidylcholine, phosphatidic acid, diglyceride, lysophosphatidic acid, and arachidonic acid. Researchers have long stressed the positive protective effects of PC on the heart by regulating the content of cholesterol in the body and effectively reducing the incidence of cholesterol, hyperlipidemia, and coronary heart disease. The role of PC in emulsifying and decomposing lipids can improve blood circulation, remove peroxides, reduce the retention time of fat in the inner walls of blood vessels, and promote the dissipation of atherosclerotic plaques; thus, it can prevent and treat arteriosclerosis. Our results showed that PC levels are decreased in patients with AS and that PC (16:0e/26:4) and PC (18:1/22:6) may be potential biomarkers of AS disease activity. Moreover, PC (16:0e/26:4) and PC (18:1/22:6) exhibited negative correlations with CRP. Based on the above results, abnormal serum lipid metabolism in patients with AS may induce cardiovascular disease. TAG (18:0/18:1/20:0), PC (16:0e/26:4), and PC (18:1/22:6) can be used as effective biomarkers for the diagnosis of AS disease.

However, the different gender distributions and ages incorporated in this study might have affected its conclusions. Due to the small number of samples used in this study, a larger sample is still needed to verify the results. Furthermore, the AS patients in this study were not graded and abnormal lipid metabolism could be observed more obviously. Since ultrahigh-performance liquid chromatography coupled with Q-Exactive spectrometry is an expensive and time-consuming process, the analysis of lipid metabolites will not be available to clinicians in everyday clinical practice; as such, they will be unable improve to early and accurate diagnoses.

In conclusion, a nontargeted HPLC-QE serum lipid method was applied to investigate the lipid characteristics of patients with AS and HCs. Consequently, it was shown that TAG (18:0/18:1/20:0), PC (16:0e/26:4), and PC (18:1/22:6) reflect the dynamic processes related to lipid levels in AS. These three lipids and their combinations can be used as potential biomarkers for the diagnosis of AS and to distinguish different degrees of the disease [31]. Understanding the changes in lipids in AS patients is an essential step in achieving proper cardiovascular risk management. 

## Figures and Tables

**Table 1:** Clinical characteristics of the subjects.

Characteristic	AS (n = 115)	HCs (n = 108)
**Basic characteristics**		
Male	71	33
Female	44	75
Age (years), mean ± SD	42.18 ± 13.76	30.68 ± 7.53
BMI (kg/m^2^), mean ± SD	25.12 ± 3.57	21.67 ± 3.60
**Clinical variables**		
ESR (mm/h), mean (median)	26.91 (2)	18.33 (20)
CRP (mg/L), mean (median)	16.04 (8.88)	7.88 (2.87)
ALP (U/L), mean (median)	88.94 (90)	86.25 (86)
ALB (g/mL), mean (median)	41.11 (41.3)	44.02 (45.7)
ASDAS, mean ± SD	2.05 ± 0.35	—
HLA-B27	87.83%	—

BMI: body mass index; ESR: Erythrocyte sedimentation rate; CRP: C-reactive protein; ALP: alkaline phosphatase; ALB: albumin; ASDAS: ankylosing spondylitis disease activity index; HLA-B27: human leukocyte antigen-B27.

**Table 2:** Identified differential lipid metabolites between the AS and HCs.

Metabolite	^a^ VIP	^b^*p* Value	^c^ FDR	^d^ FC
DAG (16:0/18:2)	2.099	<0.001	<0.001	1.520
LPC 20:3(SN2)	1.573	0.001	<0.001	1.266
PC (16:0e/26:4)	3.123	<0.001	<0.001	0.635
PC (16:1e/18:2)	1.559	<0.001	<0.001	0.743
PC (18:1/18:2.1)	1.805	<0.001	0.001	0.814
PC (18:1/22:6)	1.924	<0.001	0.001	0.759
PC (18:1e/18:2)	2.693	<0.001	<0.001	0.745
PC (20:4e/26:4)	1.889	<0.001	<0.001	0.666
PE (16:0e/22:6)	1.589	<0.001	0.001	0.856
TAG (16:0/18:1/22:5)	2.425	<0.001	<0.001	1.426
TAG (16:0/18:2/20:4)	1.783	<0.001	<0.001	1.494
TAG (16:0/20:4/22:6)	1.864	<0.001	<0.001	1.475
TAG (18:0/18:1/18:1)	2.065	<0.001	<0.001	1.456
TAG (18:0/18:1/20:4)	3.104	<0.001	<0.001	1.726
TAG (18:1/18:2/22:5)	2.684	<0.001	<0.001	1.548

^a^ VIP was obtained from the OPLS-DA model with a threshold of 1.0. ^b^ *p* Values were obtained from one-way ANOVA. The value of ^c^ FDR was obtained from the adjusted *p* Value in the metaboanalyst 5.0 software. The value of ^d^ FC was obtained by comparing those metabolites in patients with AS with the HCs. VIP: variable importance in the projection, FC: fold change, and FDR: false discovery rate.

## Data Availability

Not applicable.

## Supplementary Materials

The following supporting information can be downloaded at: https://www.mdpi.com/article/10.3390/biomedicines11010047/s1, Figure S1: Correlation analysis between PC (16:0e/26:4), PC (18:1/22:6) and CRP.

## References

[1] Chen R., Han S., Dong D., Wang Y., Liu Q., Xie W., Li M., Yao M. (2015). Serum fatty acid profiles and potential biomarkers of ankylosing spondylitis determined by gas chromatography-mass spectrometry and multivariate statistical analysis. Biomed. Chromatogr..

[2] Sundstrom B., Johansson G., Kokkonen H., Cederholm T., Wallberg-Jonsson S. (2012). Plasma phospholipid fatty acid content is related to disease activity in ankylosing spondylitis. J. Rheumatol..

[3] Nam B., Koo B.S., Lee T.H., Shin J.H., Kim J.J., Lee S., Joo K.B., Kim T.H. (2021). Low BASDAI score alone is not a good predictor of anti-tumor necrosis factor treatment efficacy in ankylosing spondylitis: A retrospective cohort study. BMC Musculoskelet. Disord..

[4] de Vlam K. (2010). Soluble and tissue biomarkers in ankylosing spondylitis. Best Pract. Res. Clin. Rheumatol..

[5] Garrett S., Jenkinson T., Kennedy L.G., Whitelock H., Gaisford P., Calin A. (1994). A new approach to defining disease status in ankylosing spondylitis: The Bath Ankylosing Spondylitis Disease Activity Index. J. Rheumatol..

[6] Lukas C., Landewe R., Sieper J., Dougados M., Davis J., Braun J., Van Der Linden S., Van Der Heijde D., Assessment of SpondyloArthritis International Society (2009). Development of an ASAS-endorsed disease activity score (ASDAS) in patients with ankylosing spondylitis. Ann. Rheum. Dis..

[7] Chandra P.E., Sokolove J., Hipp B.G., Lindstrom T.M., Elder J.T., Reveille J.D., Eberl H., Klause U., Robinson W.H. (2011). Novel multiplex technology for diagnostic characterization of rheumatoid arthritis. Arth. Res. Ther..

[8] Reveille J.D. (2015). Biomarkers for diagnosis, monitoring of progression, and treatment responses in ankylosing spondylitis and axial spondyloarthritis. Clin. Rheumatol..

[9] Fischer R., Trudgian D.C., Wright C., Thomas G., Bradbury L.A., Brown M.A., Bowness P., Kessler B.M. (2012). Discovery of candidate serum proteomic and metabolomic biomarkers in ankylosing spondylitis. Mol. Cell. Proteom..

[10] Chen C., Rong T., Li Z., Shen J. (2019). Noncoding RNAs Involved in the Pathogenesis of Ankylosing Spondylitis. Biomed. Res. Int..

[11] Nicholson J.K., Holmes E., Kinross J.M., Darzi A.W., Takats Z., Lindon J.C. (2012). Metabolic phenotyping in clinical and surgical environments. Nature.

[12] Stoll M.L., Kumar R., Lefkowitz E.J., Cron R.Q., Morrow C.D., Barnes S. (2016). Fecal metabolomics in pediatric spondyloarthritis implicate decreased metabolic diversity and altered tryptophan metabolism as pathogenic factors. Genes Immun..

[13] Wang W., Yang G.-J., Zhang J., Chen C., Jia Z.-Y., Li J., Xu W.-D. (2016). Plasma, urine and ligament tissue metabolite profiling reveals potential biomarkers of ankylosing spondylitis using NMR-based metabolic profiles. Arth. Res. Ther..

[14] He Z., Wang M., Li H., Wen C. (2019). GC-MS-based fecal metabolomics reveals gender-attributed fecal signatures in ankylosing spondylitis. Sci. Rep..

[15] Machado P., Landewe R., Lie E., Kvien T.K., Braun J., Baker D., Van Der Heijde D., Assessment of SpondyloArthritis International Society (2011). Ankylosing Spondylitis Disease Activity Score (ASDAS): Defining cut-off values for disease activity states and improvement scores. Ann. Rheum. Dis..

[16] Machado P.M., Landewe R., Heijde D.V., Assessment of SpondyloArthritis International Society (2018). Ankylosing Spondylitis Disease Activity Score (ASDAS): 2018 update of the nomenclature for disease activity states. Ann. Rheum. Dis..

[17] Van Der Heijde D., Braun J., Deodhar A., Baraliakos X., Landewe R., Richards H.B., Porter B., Readie A. (2019). Modified stoke ankylosing spondylitis spinal score as an outcome measure to assess the impact of treatment on structural progression in ankylosing spondylitis. Rheumatology.

[18] Lu J., Guo Y., Lu Y., Ji W., Lin L., Qian W., Chen W., Wang J., Lv X., Ke M. (2021). Untargeted lipidomics reveals specific lipid abnormalities in Sjogren’s syndrome. Rheumatology.

[19] Ferreira H.B., Pereira A.M., Melo T., Paiva A., Domingues M.R. (2019). Lipidomics in autoimmune diseases with main focus on systemic lupus erythematosus. J. Pharm. Biomed. Anal..

[20] Diab J., Hansen T., Goll R., Stenlund H., Ahnlund M., Jensen E., Moritz T., Florholmen J., Forsdahl G. (2019). Lipidomics in Ulcerative Colitis Reveal Alteration in Mucosal Lipid Composition Associated with the Disease State. Inflamm. Bowel Dis..

[21] Hu C., Wang M., Han X. (2017). Shotgun lipidomics in substantiating lipid peroxidation in redox biology: Methods and applications. Redox Biol..

[22] Gao P., Lu C., Zhang F., Sang P., Yang D., Li X., Kong H., Yin P., Tian J., Lu X. (2008). Integrated GC-MS and LC-MS plasma metabonomics analysis of ankylosing spondylitis. Analyst.

[23] Atzeni F., Nucera V., Galloway J., Zoltan S., Nurmohamed M. (2020). Cardiovascular risk in ankylosing spondylitis and the effect of anti-TNF drugs: A narrative review. Expert Opin. Biol. Ther..

[24] Mathieu S., Gossec L., Dougados M., Soubrier M. (2011). Cardiovascular profile in ankylosing spondylitis: A systematic review and meta-analysis. Arthritis Care Res..

[25] Exarchou S., Lie E., Lindstrom U., Askling J., Forsblad-d'Elia H., Turesson C., Kristensen L.E., Jacobsson L.T. (2016). Mortality in ankylosing spondylitis: Results from a nationwide population-based study. Ann. Rheum. Dis..

[26] Haroon N.N., Paterson J.M., Li P., Inman R.D., Haroon N. (2015). Patients with Ankylosing Spondylitis Have Increased Cardiovascular and Cerebrovascular Mortality: A Population-Based Study. Ann. Intern. Med..

[27] Papagoras C., Voulgari P.V., Drosos A.A. (2013). Atherosclerosis and cardiovascular disease in the spondyloarthritides, particularly ankylosing spondylitis and psoriatic arthritis. Clin. Exp. Rheumatol..

[28] Serdaroglu Beyazal M., Erdogan T., Turkyilmaz A.K., Devrimsel G., Cure M.C., Beyazal M., Sahin I. (2016). Relationship of serum osteoprotegerin with arterial stiffness, preclinical atherosclerosis, and disease activity in patients with ankylosing spondylitis. Clin. Rheumatol..

[29] Tada H., Kawashiri M.A., Konno T., Nohara A., Inazu A., Mabuchi H., Yamagishi M., Hayashi K. (2016). Prevalence, clinical features, and prognosis of patients with extremely low high-density lipoprotein cholesterol. J. Clin. Lipidol..

[30] Nordestgaard B.G. (2016). Triglyceride-Rich Lipoproteins and Atherosclerotic Cardiovascular Disease: New Insights From Epidemiology, Genetics, and Biology. Circ. Res..

[31] Li Z.J., Gu W.J., Qin B., Wang Y.Z., Liu S.J., Wang Z.Q., Ji W. (2022). Untargeted Lipidomics Reveals Specific Lipid Abnormalities in Ankylosing Spondylitis.

