# Supplementary material for: Untargeted Lipidomics Reveals Characteristic Biomarkers in Patients with Ankylosing Spondylitis Disease
Source: Biomedicines. 2022 Dec 25;11(1):47. doi: 10.3390/biomedicines11010047 (PMC9855684; doi:10.3390/biomedicines11010047)
Supplement: Supplementary file 1 [file biomedicines-11-00047-s001.zip › biomedicines-2094523-supplementary.pdf]

Supplementary material

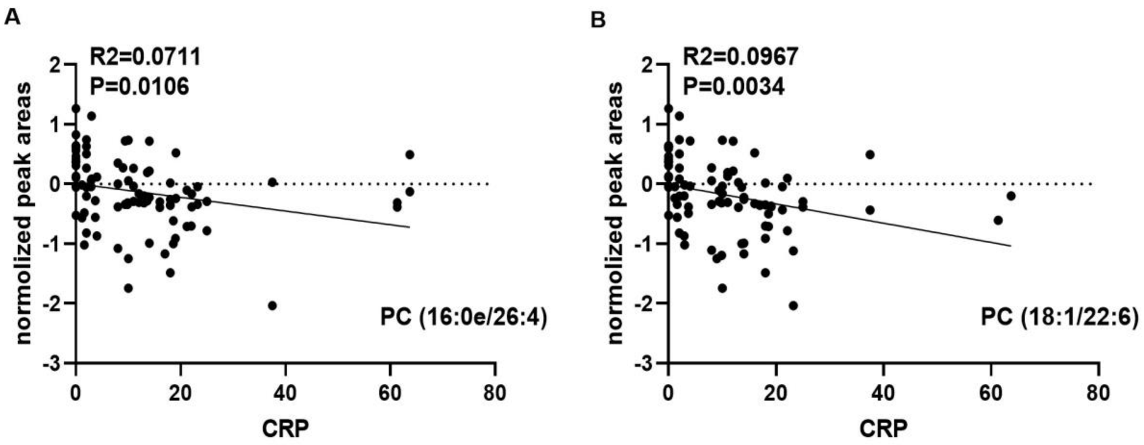

Figure S1. Correlation analysis between PC(16:0e/26:4), PC(18:1/22:6) and CRP. **(A)**Spearman correlation analysis between PC(16:0/26:4) and CRP. **(B)** Spearman correlation analysis between PC(18:1/22:6) and CRP. .
